# Cyclodextrin-Based [1]Rotaxanes on Gold Nanoparticles

**DOI:** 10.3390/ijms130810132

**Published:** 2012-08-14

**Authors:** Liangliang Zhu, Hong Yan, Yanli Zhao

**Affiliations:** 1 Division of Chemistry and Biological Chemistry, School of Physical and Mathematical Sciences, Nanyang Technological University, 21 Nanyang Link, Singapore 637371, Singapore; E-Mails: zlzhu@ntu.edu.sg (L.Z.); yanhong@ntu.edu.sg (H.Y.); 2 School of Materials Science and Engineering, Nanyang Technological University, 50 Nanyang Avenue, Singapore 639798, Singapore

**Keywords:** azobenzene, complexation, cyclodextrin, gold nanoparticle, [1]rotaxane

## Abstract

Transformation of mechanically interlocked molecules (e.g., rotaxanes and catenanes) into nanoscale materials or devices is an important step towards their real applications. In our current work, an azobenzene-modified β-cyclodextrin (β-CD) derivative that can form a self-inclusion complex in aqueous solution was prepared. The self-included β-CD derivative was then functionalized onto a gold nanoparticle (AuNP) surface via a ligand-exchange reaction in aqueous solution, leading to the formation of AuNP-[1]rotaxane hybrids. Corresponding non-self-included β-CD derivative functionalized AuNPs were also developed in a DMF/H_2_O mixture solution for control experiments. These hybrids were fully characterized by UV-vis and circular dichroism spectroscopies, together with transmission electron microscopy (TEM). The competitive binding behavior of the hybrids with an adamantane dimer was investigated.

## 1. Introduction

Mechanically interlocked molecules (MIMs) have attracted considerable attention for years as these charming systems present great potential for applications in molecular switches and machines [1–4]. Rotaxanes are one of the primary prototypes of the interlocked molecules, which consist of one or more macrocyclic components threaded by axle component(s) and trapped by bulky stoppers [5–7]. Among a variety of rotaxane-based topologies, [1]rotaxanes, whose macrocyclic rings and axle components covalently link together, stand out on account of their challenging construction and unique functions [8–10]. In order to achieve an integral device function using MIMs, much effort has been devoted to introducing these molecules onto surfaces [11,12]. The construction of [1]rotaxanes on gold nanoparticles (AuNPs), however, has not been reported to the best of our knowledge. Surface functionalization of AuNPs is a hot research topic in consequence of the tunable, fascinating physical, optical, and chemical properties of AuNPs [13,14]. Thus, in our recent research, we developed a novel type of AuNPs functionalized with β-cyclodextrin (β-CD) based on [1]rotaxanes.

**Scheme 1:**
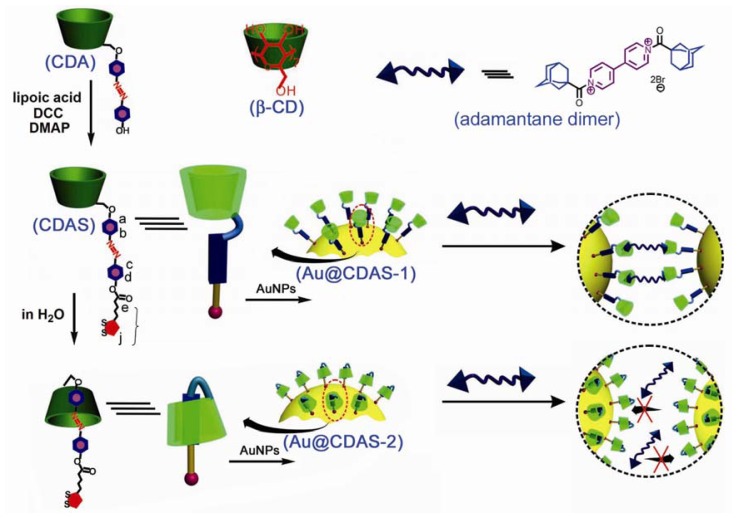
Illustration for the constructions of non-self-included β-CD derivative functionalized AuNPs (Au@CDAS-1) and β-CD-based [1]rotaxane functionalized AuNPs (Au@CDAS-2).

A crucial step to construct a [1]rotaxane is first how to fabricate a self-inclusion complex [15,16]. Herein, we synthesized a β-CD derivative CDA, which comprises an azobenzene unit directly linked to a 6-position of the β-CD ring (Scheme 1). We chose such a design because the azobenzene unit of CDA can be readily encapsulated by an intramolecular β-CD cavity in aqueous solution, rather than by an intermolecular β-CD cavity in accordance with the literature [15]. In addition, the self-inclusion process can take place even in a long chain group-substituted β-CD derivative [16]. Thus, a 1,2-dithiolane unit for binding to the AuNP surface was further introduced onto CDA via esterification between lipoic acid and the phenolic hydroxyl group of CDA, leading to the formation of the dithiolane-terminated β-CD derivative CDAS. The [1]rotaxane topology was generated through self-assembling the 1,2-dithiolane unit of the self-included CDAS onto the AuNP surface (Scheme 1).

## 2. Results and Discussion

### 2.1. Self-Inclusion of CDAS in an Aqueous Environment

**Figure 1:**
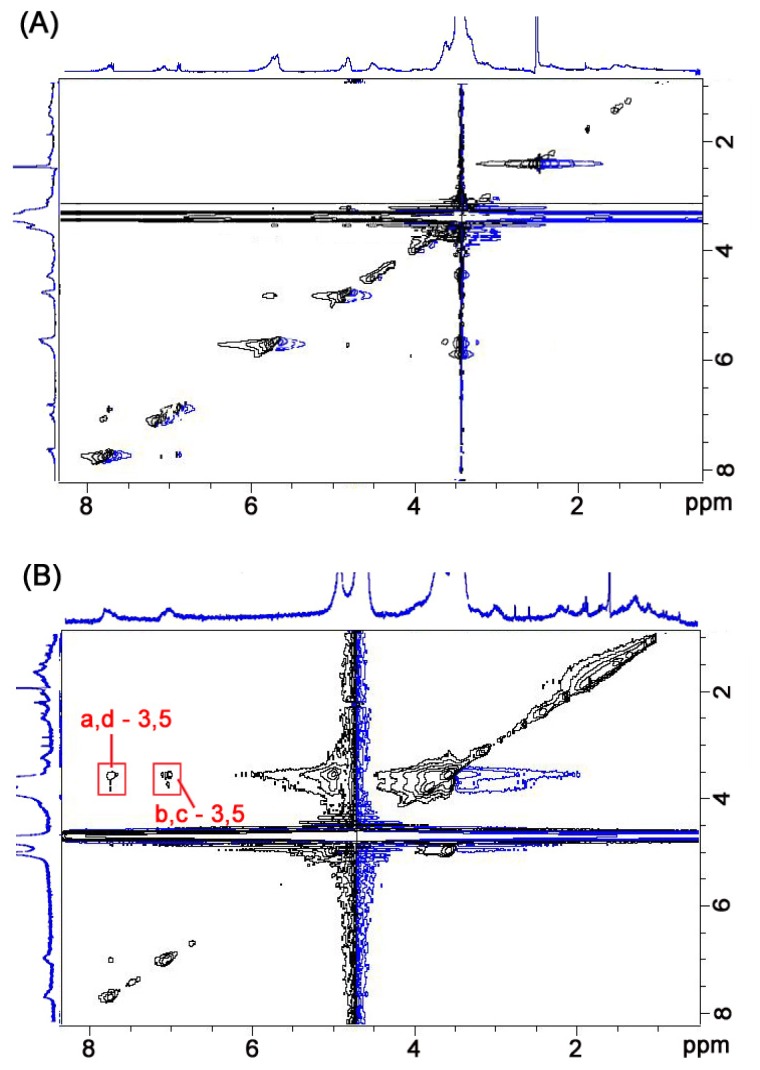
The ^1^H NMR NOESY spectra (400 MHz) of CDAS in (**A**) DMSO-*d*_6_ and (**B**) D_2_O. The labels for protons in azobenzene-substituted β-cyclodextrin (CDAS) are shown in Scheme 1.

The synthesis and characterization details of the compound CDAS are shown in the experimental section. The solubility of this compound varies in a variety of solvents. CDAS is soluble in some polar organic solvents such as DMF and DMSO, but exhibits a relatively low solubility in H_2_O. The ^1^H NOESY NMR spectra of CDAS in DMSO-*d*_6_ and in D_2_O are shown in Figure 1A,B, respectively. We cannot find any NOE cross signals in Figure 1A although the proton resonances of CDAS can be well assigned. In contrast, NOE cross signals between the H_a–d_ protons of the azobenzene unit and the H_3/5_ protons within the β-CD cavity are found as shown in Figure 1B. There are no NOE cross signals between the protons of lipoic acid unit and the internal ones of the β-CD cavity. These observations indicate that the azobenzene unit of CDAS is exclusively included within the β-CD cavity in aqueous solution. Conversely, this molecule adopts a non-inclusion conformation in organic solvents such as DMSO.

**Figure 2:**
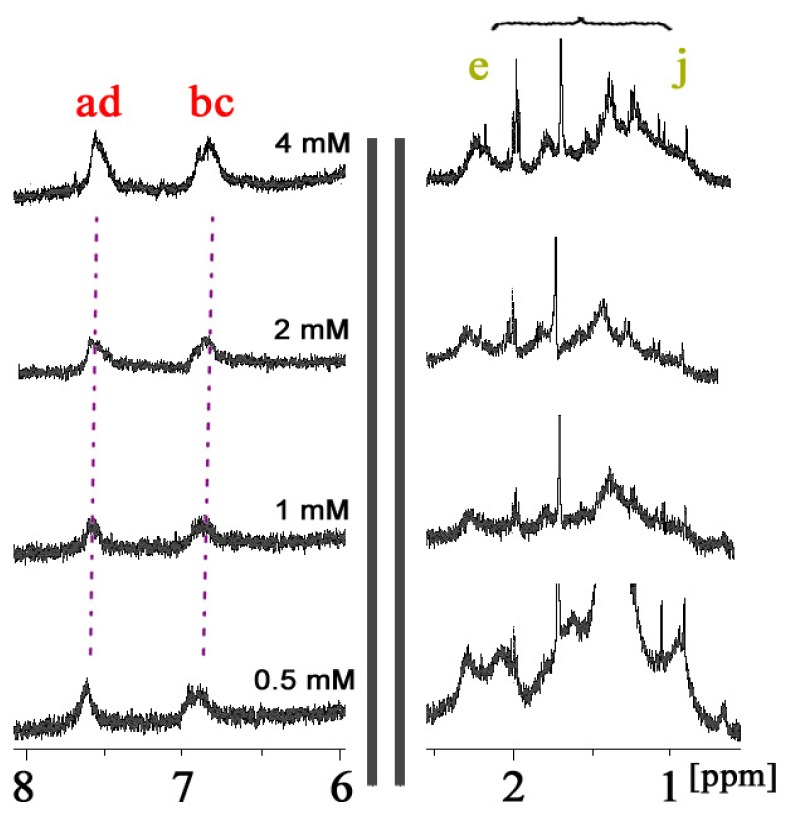
^1^H NMR spectra of CDAS in D_2_O at 298 K at different concentrations.

In order to confirm whether such complexation is self-inclusion behavior or not, the conformational study of CDAS with different concentrations was carried out in aqueous solution. The ^1^H NMR spectrum of CDAS in D_2_O does not present any concentration-dependent effect under the investigated concentration range (0.5–4.0 mM). The azobenzene protons H_a–d_ only present a very small downfield shift (less than 0.1 ppm) when decreasing the concentration of CDAS (Figure 2), simply because of natural dethreading. The protons of the lipoic acid unit also do not show obvious chemical shifts at different concentrations. These observations indicate that there is no significant conformational change of CDAS under the concentration range [17].

**Figure 3:**
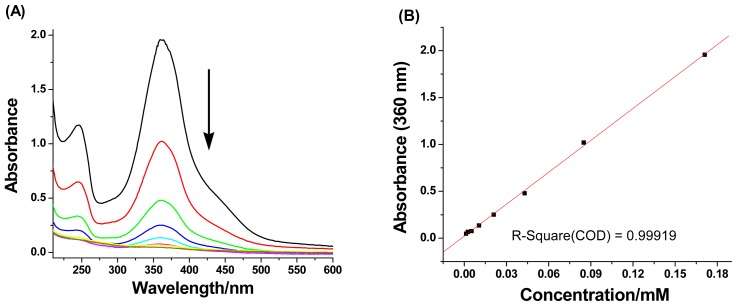
(**A**) UV-vis absorbance of CDAS in H_2_O at 298 K under different concentrations, and (**B**) Plot of the UV-vis absorbance against the concentration. The absorbance of CDAS was measured at *λ*_max_ = 360 nm.

At lower concentration range, the ^1^H NMR technique cannot be used for evaluation because of weak proton signals. Hence, we employed UV-vis absorption measurements for further examination. The absorbance of CDAS at *λ*_max_ = 360 nm was recorded (Figure 3) under the concentration range from 0.171 mM to 0.0013 mM. Plotting the absorbance against the concentration provides (Figure 3B) a linear line (*R*^2^ = 0.99919), indicating that CDAS undergoes a unimolecular conformational exchange, which is not affected by changes in concentration [18]. Thus, we can conclude that CDAS prefers to form a self-included complex in aqueous solution, rather than dimers or high-order oligomers.

### 2.2. Construction of [1]Rotaxanes on AuNPs

**Figure 4:**
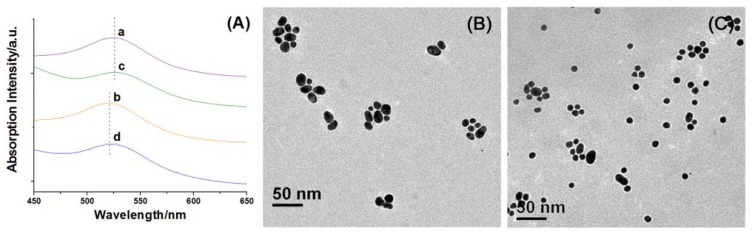
(**A**) Normalized absorption spectra of citrate-stabilized AuNPs in aqueous solution with addition of (a) DMF, (b) H_2_O, (c) DMF solution of CDAS, and (d) aqueous solution of CDAS; (**B**) TEM image of Au@CDAS-1; (**C**) TEM image of Au@CDAS-2.

Non-self-included CDAS functionalized AuNPs (Au@CDAS-1) and [1]rotaxane decorated AuNPs (Au@CDAS-2) were prepared by ligand-exchange reactions (see the Experimental Section for details). The original citrate-stabilized AuNPs (average diameter of 15 nm) present a characteristic surface plasmon resonance (SPR) band at 520 nm in aqueous solution. When the AuNP aqueous solution is mixed with an equal volume of DMF, the SPR band slightly red-shifts to 523 nm. The SPR band around 523 nm is not affected in the presence of CDAS. When adding an equal volume of DMF solution containing CDAS, the SPR band of AuNPs is almost the same as when just adding pure DMF (curves a and c in Figure 4A). Similarly, the SPR band of AuNPs remains unchanged by adding either aqueous solution of CDAS or same amount of pure H_2_O (curves b and d in Figure 4A). These results indicate that the introduction of CDAS does not cause aggregation of AuNPs.

These AuNP hybrids (Au@CDAS-1 and Au@CDAS-2) can be facilely isolated from the ligand solutions by centrifugation (6000 rpm, 60 min). When they were re-dispersed in H_2_O by sonication, stable solutions with reddish purple color could be obtained. TEM technique was employed to investigate the morphologies of the hybrids. From the TEM images (Figure 4B,C), we can observe that the two hybrids are mainly monodispersed. These characterizations demonstrate that both Au@CDAS-1 and Au@CDAS-2 are formed by individual CDAS molecules attached onto the AuNP surface rather than a cross-linked inter-particle process (see the schematic representations of Au@CDAS-1 and Au@CDAS-2 in Scheme 1).

**Figure 5:**
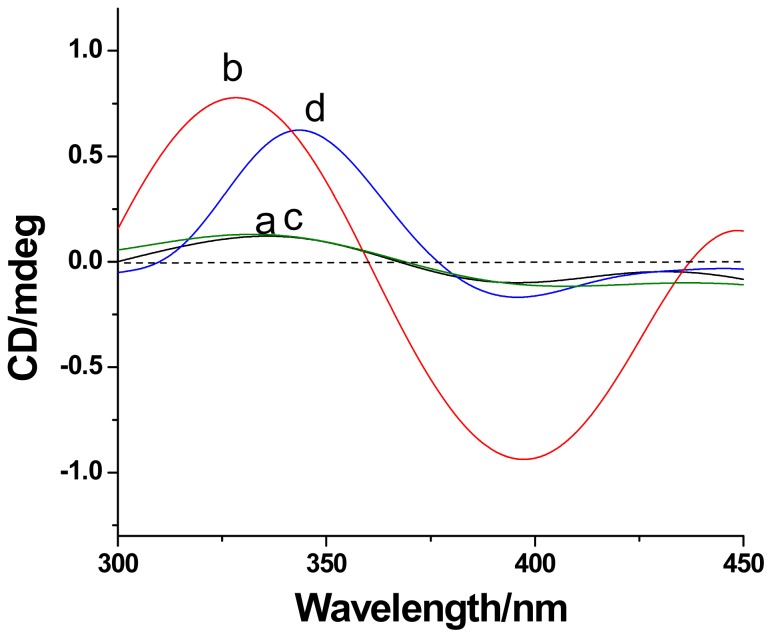
The induced circular dichroism (ICD) spectra of CDAS in (**a**) DMF and (**b**) H_2_O, (**c**) Au@CDAS-1 in DMF, and (**d**) Au@CDAS-2 in H_2_O at 298 K.

Induced circular dichroism (ICD) is an important technique for the characterization of cyclodextrin based (supra)molecular systems [19]. Non-self-included CDAS in DMF shows no significant ICD signal (curve a of Figure 5). The self-complexation of the azobenzene unit into its own β-CD cavity in aqueous solution (curve b of Figure 5) leads to a positive cotton effect at about 330 nm and a negative one at around 400 nm. The positive ICD signal can be attributed to the electronic transition π–π* of the azobenzene unit, which locates in the β-CD cavity and aligns parallel to the symmetric axis of the chiral host. The negative one belongs to the transition n–π* of the azobenzene unit that aligns vertical to the symmetric axis of the β-CD ring [20]. Similar to the case of CDAS in DMF, no ICD signal was observed for Au@CDAS-1 in aqueous solution (curve c of Figure 5), since there is no self-inclusion behavior on the AuNP surface. In contrast, a positive Cotton effect around 350 nm can be found from Au@CDAS-2 in aqueous solution (curve d of Figure 5), meaning that the azobenzene unit was indeed included into its own β-CD cavity to form [1]rotaxane on the AuNP surface. However, the negative Cotton effect of Au@CDAS-2 at 400 nm is weak, indicating that the azobenzene unit in the β-CD cavity becomes more acclivitous after the self-included CDAS grafts onto the AuNP surface.

### 2.3. Competitive Binding with Adamantane Dimer

**Figure 6:**
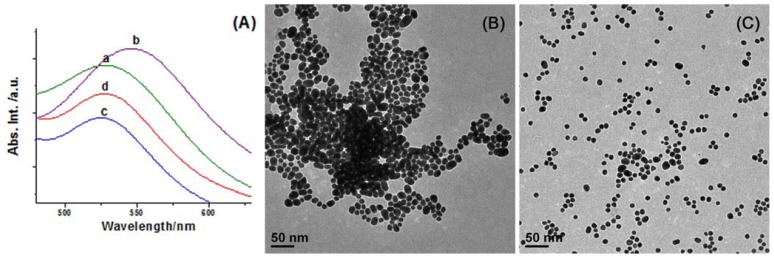
(**A**) Normalized absorption spectra (SPR band) of Au@CDAS-1 in aqueous solution (a) before and (b) after the addition of excess amount of adamantane dimer, and Au@CDAS-2 in aqueous solution (c) before and (d) after the addition of excess amount of adamantane dimer; (**B**) TEM image of Au@CDAS-1 after the addition of excess amount of adamantane dimer; (**C**) TEM image of Au@CDAS-2 after the addition of excess amount of adamantane dimer.

As [1]rotaxane is of a closed structure whose macrocyclic component is occupied by the axle itself it thus loses the binding ability towards additional guests. On the contrary, a competitive binding process can take place in a non-included case. To further investigate the difference between two AuNP hybrids, we introduced an adamantane dimer (see the chemical structure in Scheme 1) to study the competitive binding behavior, since the association constant between the β-CD ring and adamantane derivatives is relatively high (normally more than 10^5^ M^−1^). The SPR band of Au@CDAS-1 in aqueous solution presents an apparent red-shift (ca. 20 nm) after adding excess amount of adamantane dimer (see curves a and b in Figure 6A), indicating that the two adamantane groups in the dimer are encapsulated by the β-CD rings from different nanoparticles to form inter-particle clusters (see the schematic representation in Scheme 1). However, the SPR band of Au@CDAS-1 in aqueous solution shows no shift after adding excess amount of adamantane dimer (see curves c and d in Figure 6A), certifying a closed [1]rotaxane conformation in Au@CDAS-2 where the β-CD ring is mechanically occupied by the azobenzene unit and cannot bind adamantane dimer (see the schematic representation in Scheme 1). The TEM images (Figure 6B,C) show significant nanoparticle aggregates and mono-dispersed nanoparticles for Au@CDAS-1 and Au@CDAS-2 after the addition of excess amount of adamantane dimer, respectively, further confirming the above mentioned conclusion.

### 2.4. Photoisomerization of [1]Rotaxanes on AuNPs

**Figure 7:**
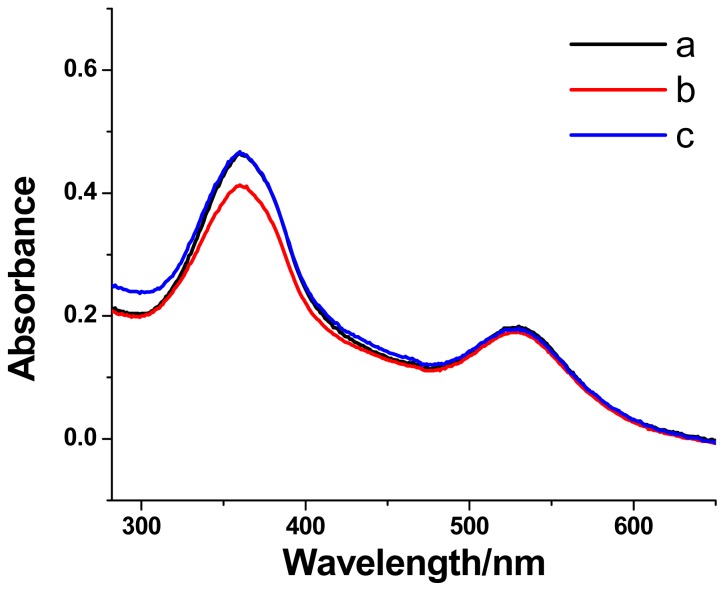
The absorption spectra of the AuNP-[1]rotaxane hybrid Au@CDAS-2 (a) in initial state, (b) after UV light irradiation at 365 nm, and (c) after visible light irradiation.

Normally, azobenzene compounds adopt a *trans*-conformation and can undergo the *trans*-to-*cis* photoisomerization under UV light irradiation at 365 nm. When the *trans*-azobenzene unit transforms to its *cis*-form, the *cis*-azobenzene unit will be excluded from the β-CD cavity due to the weak binding constant between the *cis*-azobenzene unit and the β-CD ring. The photoisomerization of the azobenzene unit in Au@CDAS-2 was also explored. A reversible photoisomerization process can be evidenced from the UV-vis investigations (Figure 7). The reduction of the maximum absorption band around 360 nm from the azobenzene unit upon UV light irradiation at 365 nm, and the recovery of the band under visible light irradiation were observed, indicating the *trans*-*cis* photoisomerization of the azobenzene unit in Au@CDAS-2.

## 3. Experimental Section

### 3.1. General

^1^H NMR, ^13^C NMR, and ^1^H NOESY NMR spectra were recorded on a Bruker BBFO-400 spectrometer. High-resolution mass spectrometry (HR-MS) was performed on a Waters Q-tof Premier MS spectrometer. Absorption spectra were recorded on a Shimadzu UV-3600 UV-Vis-NIR spectrophotometer using a 1 cm quartz cell, and circular dichroism spectra were recorded on a Jasco J-810 CD spectrophotometer using a 1 cm quartz cell. Transmission electron microscopy (TEM) images were collected on a JEM-1400 (JEOL) operated at 100–120 kV. Melting points were determined by using an OptiMelt automated melting point system.

### 3.2. Materials

β-CD, dicyclohexyl carbodiimide (DCC), 4-dimethylamino pyridine (DMAP), hydrogen tetrachloroaurate, (±)-α-lipoic acid, 4-nitrophenol, sodium citrate, and *p*-toluenesulfonyl chloride were purchased from Sigma Aldrich and used as received. The vials, stirring bars, and cuvettes were pretreated with aqua regia before use.

### 3.3. Synthesis of Mono-(6-*O*-p-Toluenesulfonyl)-β-CD

This compound was prepared according to a reported procedure [21].

### 3.4. Synthesis of 4-((4-Hydroxyphenyl)diazenyl)phenol

This compound was prepared according to our previous work [14].

### 3.5. Synthesis of Mono-(6-*O*-4-((4-Hydroxyphenyl)diazenyl)phenyl)-β-CD (CDA)

To a solution of DMF (15 mL) containing mono-(6-*O*-*p*-toluenesulfonyl)-β-CD (1 g, 0.775 mmol) was added 4-((4-hydroxyphenyl)diazenyl)phenol (1 g, 4.67 mmol). The mixture as a solution was stirred at 90 °C under argon for 3 days. The solution was poured into THF (80 mL), and the precipitate formed was collected by filtration to give a crude powder. The product was then applied to silica gel chromatography (*n*-butanol:EtOH:H_2_O = 5:4:3) to give a pure compound (471 mg, 45.7%). Melting point > 250 °C. ^1^H NMR (400 MHz, DMSO-*d*_6_, 298 K, TMS): *δ* = 7.78 (d, *J* = 8.4 Hz, 2H), 7.75 (d, *J* = 8.4 Hz, 2H), 7.10 (d, *J* = 8.4 Hz, 2H), 6.94 (d, *J* = 8.4 Hz, 2H), 5.66~5.81 (m, 14H), 4.78~4.95 (m, 7H), 4.38~4.52 (m, 5H), 4.25~4.35 (m, 2H), 3.99 (m, 1H), 3.15~3.75 (m). ^13^C NMR (400 MHz, DMSO-*d*_6_, 298K, TMS): *δ* = 160.95, 160.88, 146.68, 145.69, 125.27, 124.63, 116.32, 115.47, 102.42, 82.02, 73.54, 72.91, 72.52, 60.77, 60.40. HR-MS (ESI): calcd. for C_54_H_79_N_2_O_36_ *m*/*z* = 1331.4413, found *m*/*z* = 1331.4380.

### 3.6. Synthesis of CDAS

A mixture of CDA (0.9 g, 0.677 mmol), (±)-α-lipoic acid (0.97 g, 4.74 mmol), DCC (0.14 g, 0.677 mmol), and DMAP (5.9 mg, 0.05 mmol) in anhydrous DMF (10 mL) was stirred at 90 °C for 36 h. The solution was poured into THF (50 mL), and the precipitate formed was collected by filtration to give a crude powder. The powder was washed with deionized H_2_O and then applied to silica gel chromatography (*n*-butanol:EtOH:H_2_O = 5:4:3) to give a pure compound (295 mg, 29.1%). Melting point > 250 °C. ^1^H NMR (400 MHz, DMSO-*d*_6_, 298 K, TMS): *δ* = 7.79 (d, *J* = 8.4 Hz, 2H), 7.75 (d, *J* = 8.4 Hz, 2H), 7.11 (d, *J* = 8.4 Hz, 2H), 6.92 (d, *J* = 8.4 Hz, 2H), 5.64~5.89 (m, 14H), 4.78~4.95 (m, 8H), 4.25~4.52 (m, 7H), 4.05 (m, 2H), 2.38 (m, 1H), 1.90 (m, 1H), 1.69 (m, 1H), 1.53 (m, 3H), 1.35 (m, 2H). ^13^C NMR (400 MHz, DMSO-*d*_6_, 298K, TMS): *δ* = 173.05, 160.96, 160.76, 146.70, 145.75, 124.82, 124.37, 116.32, 115.42, 102.44, 82.04, 73.53, 72.92, 72.52, 60.26, 56.60, 38.65, 34.59, 34.12, 28.66, 24.45. HR-MS (ESI): calcd. for C_62_H_91_N_2_O_37_S_2_ *m*/*z* = 1519.4742, found *m*/*z* = 1519.4702.

### 3.7. Citrate-Stabilized AuNPs with an Average Diameter of 15 nm

Citrate-stabilized AuNPs with an average diameter of 15 nm were prepared according to a literature report [22].

### 3.8. Preparations of Au@CDAS-1 and Au@CDAS-2

Two hybrids were prepared by the ligand-exchange reaction. Typically, CDAS dissolved in DMF (2 mL, 0.2 mM) or deionized H_2_O (2 mL, 0.2 mM) was placed in a 4-mL cleaned vial. Citrate-stabilized AuNP aqueous solution (1 mL, 4.5 nM calculated by the gold spheres) was added dropwise to the above solution with stirring for 6 h. Au@CDAS-1 and Au@CDAS-2 were generated as stable substances and isolated by centrifugation (13,500 rpm, 15 min), respectively. Then, the precipitates were washed with H_2_O till no UV absorption could be detected from the eluent. The hybrid solids can be dried in vacuo or re-dispersed in H_2_O for subsequent investigations.

## 4. Conclusions

β-cyclodextrin-based [1]rotaxane functionalized gold nanoparticles were developed by introducing azobenzene-substituted β-cyclodextrin (CDAS) onto the surface of gold nanoparticles via a ligand-exchange reaction. The key step in the synthetic strategy is the preparation of self-included CDAS in H_2_O as an intermediate. In this process, an aqueous solution is beneficial to the formation of the AuNP-[1]rotaxane hybrid, whereas non-self-included CDAS functionalized gold nanoparticles can be produced in a DMF/H_2_O mixture solution. The combination of molecular machines and metal nanoparticles into single entities may lead to new applications in nanoscale digital information processing.
